# Dystonia and the Role of Deep Brain Stimulation

**DOI:** 10.5402/2011/193718

**Published:** 2011-04-13

**Authors:** Thomas L. Ellis

**Affiliations:** Wake Forest School of Medicine, Wake Forest University, Medical Center Boulevard, Winston Salem, NC 27157, USA

## Abstract

Dystonia is a painful, disabling disease whose cause in many cases remains unknown. It has historically been treated with a variety methodologies including baclofen pumps, Botox injection, peripheral denervation, and stereotactic surgery. Deep brain stimulation (DBS) is emerging as a viable treatment option for selected patients with dystonia. Results of DBS for dystonia appear to be more consistently superior in patients with primary versus secondary forms of the disorder. Patients with secondary dystonia, due to a variety of causes, may still be candidates for DBS surgery, although the results may not be as consistently good. The procedure is relatively safe with a small likelihood of morbidity and mortality. A randomized trial is needed to determine who are the best patients and when it is best to proceed with surgery.

## 1. Introduction

Dystonia is a painful and severely disabling disease, whose cause is unknown, but is believed to be disorder of the basal ganglia. For patients with the rare, generalized form, treatment options are limited, but include baclofen pumps, peripheral denervation surgery, and stereotactic surgery. While lesioning the thalamus was originally the preferred surgical treatment option for dystonia [1, 2], recent interest has shifted the focus onto the globus pallidus internus (GPi). This derived mainly from the noteworthy improvement in dystonic symptoms associated with Parkinson's disease which have been demonstrated after pallidotomy [3–5]. Although there are multiple case reports of dystonia being successfully treated with pallidotomy [6–10], there can be significant side effects, and the benefits may not be durable [2]. With the development of DBS over the last decade, the surgery has become safer, and the side effects have become easier to control. Moreover, as the disease progresses, stimulation can be varied to improve outcomes. 

Variable muscle groups may be involved to a differing extent and severity. The focal dystonias (e.g., Torticollis, Writer's cramp) involve a single body region while the generalized dystonias involve wide spread axial and limb muscles. The dystonias may be classified by three criteria: etiology, age of onset (early versus late), and anatomical distribution (focal, segmental, multifocal, and generalized). With regard to etiology the classification includes primary (idiopathic) and secondary (symptomatic). Accurate estimates about the prevalence of dystonia in the general population are often confounded by confusion regarding the diagnosis [11]. Nutt et al. estimated the prevalence to be 29.5 per 100,000 for focal dystonias and 3.4 for 100,000 per generalized dystonias [12]. A more recent collaborative epidemiologically study in Europe estimated an annual prevalence of 15.2 per 100,000 with the majority cases consisting of focal dystonias [13]. The age of onset of primary dystonia is bimodal in distribution with peak incidences occurring at 9 (early onset) and 45 (late onset) years of age [11]. 

The exact pathophysiology of the dystonias remains unkown. Neurophysiological studies suggest that a dysfunction within the basal ganglia leads to loss of cortical inhibition, resulting in excessive cortical activity, thus producing the abnormal movements [14]. Only a minority of patients with symptomatic generalized dystonia—namely, those with Wilson's disease, psychogenic dystonia, and dopa responsive dystonia (DRD)—have specific treatment options [15]. As a result, the evaluation of all adolescents and children with new onset dystonia should include a levodopa trial and should rule out Wilson's disease. The characteristic feature of DRD is a significant, sustained response of the Parkinsonian and dystonic symptoms to low-dose levodopa. Therefore, all pediatric cases of dystonia without a known etiology should be treated with levodopa in the early stages. For the remaining patients with dystonia the goal of treatment is to improve symptoms rather than address the underlying cause. 

Nonsurgical treatment aimed at controlling symptoms include medications and botulinum toxin. Although multiple medications have been found to produce benefit in selected patients with dystonia, no single agent can consistently relieve symptoms [16]. Botulinum toxin is currently the primary treatment for many focal dystonias including torticollis, blepharospasm, and spasmodic dysphonia [17, 18]. 

Surgery aimed at improving dystonic symptoms includes targeted procedures of both peripheral and CNS structures. Denervation procedures have been used, with some positive results, primarily in the treatment of cervical dystonia [19]. Progress in understanding the physiology of the basal ganglia, refined surgical techniques, and the discovery that pallidotomy can improve “off state” dystonia in Parkinson's disease patients have led to renewed interest in CNS procedures for the treatment of dystonia [5, 20]. Published reports have described improvement in symptoms of patients with primary generalized dystonia undergoing pallidotomy [8, 21]. The primary limitation of this approach is that unilateral pallidotomy may be insufficient to treat axial disease while bilateral pallidotomy is associated with significant risk of dysphagia, dysarthria, and cognitive dysfunction [22, 23]. Moreover, because these procedures are irreversible, it is possible that they may interfere with future treatment options which may become available with increased understanding of the neurophysiological and genetic basis of dystonia.

## 2. Deep Brain Stimulation

Deep Brain Stimulation (DBS) has the potential to overcome these hurtles. It has significant advantages over ablative procedures including the following.

Reversibility—if side effects are noted, stimulation parameters can be adjusted through programming or the stimulator turned off. This will allow patients to remain candidates for future therapies that may arise as a result of progress in molecular genetic research.

Programmability—unlike with ablative procedures, stimulation parameters can be adjusted as needed over time to improve the response and minimize the side effects. Moreover, the programmability provides a mechanism by which to increase our understanding of the physiological of dystonia and other movement disorders.

Bilateral targeting—the ability to turn to stimulators off combined with the minimal amount of tissue damage caused by the electrodes allows bilateral procedures to be performed more safely as compared to ablative procedures. 

The disadvantages of DBS include high costs, higher risk of infection, the need for periodic battery replacement, and the potential for hardware malfunction.

While there is currently no consensus as to the ideal site for DBS implantation for the treatment of dystonia, the thalamus, subthalamic nucleus, and globus pallidus have been targeted successfully [24, 26, 27]. The methodology for implanting electrodes is also variable. Target localization at some centers still includes ventriculography [26, 27], although CT and MRI are rapidly becoming more prevalent. Implantation can occur while the patient is under general anesthesia, although at most centers it is performed under local anesthetic alone. While some centers perform bilateral procedures staged over weeks or even months, others prefer to implant simultaneous bilateral electrodes. 

Coubes et al. have implanted both electrodes in a single session under general anesthesia. The targeting was performed based on MRI alone without intraoperative microelectrode recording (MER) [28]. By contrast Krauss and colleagues performed the procedure under local anesthesia, targeting the posteroventral lateral GPi using MER [29]. They subsequently perform macrostimulation with the DBS electrode in order to determine thresholds and responses prior to finalizing the electrode position.

Tagliati et al. treat medically refractory primary dystonia with bilateral DBS electrodes implanted during a single stereotactic procedure [16]. In most cases, the patients are implanted awake in order to facilitate MER. In the patients who are either too young or too severely affected by their dystonic symptoms to tolerate awake surgery, they use either dexmedetomidine hydrochloride or propofol for light sedation.

At Wake Forest University, we generally implant both electrodes in a single session to spare the dystonia patients a second frame application. We have also found that the younger patient population seen in dystonia is less likely to undergo the brain shift often seen in the older population when simultaneous burr holes are placed (unpublished data). After completing MER targeting, macrostimulation with the DBS electrode is performed to ensure that the patient does not suffer from serious side effects. Production of abnormal visual scotomata is an evidence that the electrode is too deep and may need to be withdrawn. Production of motor contractions results from an electrode that is too lateral, requiring that the electrode be moved medially. After the target is anatomically and electrophysiologically refined, the electrode is secured to the calvarium using the Navigus system. The proximal end is then tunneled in a subgaleal fashion to the parietal region. After completing one side, the same procedure is performed on the contralateral brain. The proximal end of both electrodes can be tunneled to the same parietal location. The extension sets and pulse generator(s) are then implanted under general anesthesia during a second procedure, typically 3 weeks after the electrode implantation. 

To determine whether a patient is a suitable candidate for DBS implantation, the neurologist and neurosurgeon must consider not only the motor status, but also the cognitive, physiological, and overall medical condition. The most suitable candidates appear to be those with segmental, generalized dystonia who have failed to respond to botulinum toxin injections or medication and thus suffer from continued pain and disability [30]. This screening must include a levodopa trial in order to exclude patients with DRD and should determine whether the symptom to be treated with DBS is the primary source of disability. Recent experience with DBS suggests that primary dystonia (especially the DYT1-positive) responds more favorably than secondary dystonia [16, 25, 31]. The effects of DBS on tardive dystonia and myoclonic dystonia, while encouraging, have been studied less extensively. The ideal timing of surgery is debated, but it should be performed prior to the onset of contractures of other orthopedic complications, in order to maximize neurological rehabilitation.

## 3. Clinical Results

Mundinger was the first to record the use of DBS for dystonia in 1977 [16]. He implanted 7 patients with cervical dystonia with unilateral electrodes. They subsequently underwent low frequency, intermittent stimulation of the thalamic and sub thalamic nuclei. They observed good results although the follow-up period was short. Subsequent to this, DBS for dystonia was largely abandoned until the late 1980s. In light of the favorable results achieved with pallidotomy for dystonia at that time, DBS of the GPi soon re-emerged. Early results of GPi DBS for dystonia were so encouraging that the FDA granted a humanitarian device exemption (HDE) for the Medtronic Activa DBS system for the treatment of primary generalized dystonia in April 2003. 

Patients with primary dystonia appear to respond better than those with secondary dystonia. Patients with DYT1-positive primary dystonia appear to derive the most benefit, with improvements of up to 90% on the Burke-Fahn-Marsden Dystonia Rating Scale (BFMDRS) [25, 27, 31–34]. The DYT1 form is an inherited, generalized dystonia caused by a single GAG deletion in the DYT1 gene located on chromosome 9q. This gene encodes torsin A which is a member of the family of AAA adenosine triphosphates. Although the inheritance pattern of DYT1 gene mutation is autosomal dominant, the phenotypic penetrance is 30% to 40%. The DYT1 mutation is more common in the Ashkenazi Jewish populations, but has also been identified in non-Jewish Asian, European, and North American families. Patients with DYT1-positive dystonia most commonly present in childhood with limb dystonia, which may subsequently become generalized. 

Improvements in the tonic components tend to be more gradual, and the maximal benefit from DBS may not be seen for 6–12 months. The results in patients with non-DYT1 primary dystonia, while less impressive, are still quite good [35, 36]. Positive results have also been reported in cases of familial myoclonic dystonia, an autosomal dominant disorder with onset in childhood or early adolescence. 

Reports documenting the effect of pallidal DBS on secondary dystonia have been more varied and perhaps less encouraging [25, 36, 37]. This subgroup of patients has a more heterogeneous profile with regard to etiology, clinical manifestations, and prognosis. Chang et al. reported long-term effects of bilateral pallidal DBS for patients with tardive dystonia secondary to neuroleptic medication use [38]. The mean baseline BFMDRS score was 49.7 (range 20–88). The followup lasted from 2 to 8 years during which they observed a 62% improvement in the BFMDRS. 71% of patients demonstrated sustained improvement at the time of the last follow-up. 

Speelman et al. recently reviewed the literature and found reports on 109 patients undergoing DBS for secondary dystonia [39]. In most cases, the cause was tardive dystonia (38 patients), cerebral palsy (18 patients), myoclonus-dystonias (12 patients), or neurodegeneration with brain iron accumulation (13 patients). The remaining patients were distributed under a wide variety of diagnoses ranging from rapid onset dystonia parkinsonism to postanoxic secondary generalized dystonia. The outcomes also varied widely based on the diagnosis. Of those with tardive dystonia, 27 patients (69%) experienced whether a good or very good outcome. In those patients with myoclonus-dystonia, 12 (100%) received a very good result. In patients with NBIA, 8/13 (64%) obtained either good or very good outcome after pallidal DBS electrode implantation. 

Many of the patients with secondary dystonia have other neurological disorders, including spasticity, seizures, dementia, and cerebrovascular disease, or cerebellar symptoms that may limit optimal response to surgery. Many studies report little to no benefit or even worsened outcomes after their DBS in secondary dystonia. The exception to this however may be in patients with tardive dystonia which appear to respond favorably to DBS. A normal brain MRI may be a predictor of favorable response to surgery. 

Coubes et al. have published the largest series to date of GPi DBS for dystonia [28]. They reported on their experience with 53 patients, including 15 with DYT1-positive primary dystonia, 17 with non-DYT1 primary dystonia, and 21 with secondary dystonia. Followup ranged from 6 months to 5 years. One year after surgery, the DYT1-positive patients improved an average of 71% on their clinical scores. The non-DYT1 patients improved 74% on average. Those with secondary dystonia demonstrated an average improvement of only 31%. The efficacy of stimulation did not appear to decrease with time. Complications included 1 lead fracture and 3 patients with an infection. 

 Tagliati and colleagues implanted bilateral DBS leads in the GPi of 12 patients with medically refractor primary dystonia and 3 patients with secondary dystonia [16]. Seven of the patients with primary dystonia were DYT1-positive. Two of the DYT1-positive patients had undergone multiple thalamotomy 30 years prior to DBS implantation. The 3 patients with secondary dystonia had a childhood history of encephalitis or encephalopathy. Intraoperative MER, MRI, and fluoroscopy were all used to refine the targeting of the GPi. All patients were evaluated with the BFMDRS before and after surgery. The DBS output settings were titrated upward after surgery to achieve the best clinical outcome. Optimal results were found at a high frequency (130 Hz) and with a large pulse width (210–400 microseconds). Percent changes of BFMDRS scores were calculated postoperatively at a minimum of 6 months of followup. All of the patients with primary dystonia demonstrated improvement in their BFMDRS scores (range 32–97%). The improvement was found to be progressive over time with an average improvement of 33.2% at 1 month, 47.2% at 3 months, 60.4% at 6 months, 72.9% at 1 year, and 78.9% at 2 years. Two of the DYT1-positive patients demonstrated an improvement in the BFMDRS of 77% and 95%, respectively at 3 years. The 3 patients with secondary dystonia showed an average improvement of 32.6% on their BFMDRS scores. The two DYT1-positive patients with prior thalamotomy demonstrated results comparable to those observed in the patients with secondary dystonia. Complications included 1 superficial infection requiring lead revision and 2 fractured wires.

Kumar et al. [40] and Coubes et al. [32] reported one case each of an early onset, generalized dystonia that improved significantly after DBS of the GPi. Coubes et al. subsequently reported on seven patients (6 children, 1 adult) with rapidly progressing, DYT1-positive generalized dystonia who underwent bilateral posteroventral GPi DBS implantation [32]. The mean age at surgery was 14 years 6 months (range 8–27 years). Neurological status was assessed pre- and postoperatively at selected intervals using the BMFDRS. The duration of followup was at least 1 year for all patients. Stimulation variables included high frequency (130 Hz), monopolar low voltage (1.6 V), with continuous stimulation on one or two contacts. Generalized dystonia improved gradually over 3 months. There was a progressive improvement in function with dystonic movements disappearing over time. All six children managed learned to walk without assistance. In the lone adult patient, secondary spinal and lower limb deformities limited complete recovery even though dystonia was significantly improved. Pain rapidly improved in all patients. Infection in one patient required removal of the entire DBS system with successful reimplantation 6 months later. No other adverse events were reported. They documented an improvement ranging from 60% to 100% in the BFMDS score at the one year followup. 

Yianni et al. reported their outcomes with two patients suffering from DYT1-positive generalized dystonia who underwent bilateral GPi DBS [36]. At followup times of six and twelve months, respectively, they reported improvement in the total BFMDS score of approximately 40% and 85%. This study also included 12 patients with DYT1-negative dystonia, with seven patients having a young age at disease onset. While the outcomes were highly varied, they found an average improvement of 46% in the total BFMDS score. Krauss et al. reported their experience with two patients with a DYT1-negative generalized dystonia who underwent bilateral GPi DBS. After two years of followup, they discovered an improvement in the BFMDS motor score of 70% to 80%. 

More recently, Haridas et al. reported their result on 22 consecutive patients <21 years of age with primary generalized dystonia who underwent DBS implantation [41]. The followup was quite good with all 22 patients reaching 1-year followup, 14 reaching 2 years, and 11 reaching 33 years. The motor subscores of the BFMDRS improved by 84%, 93%, and 94% at these follow-up points. Comparable improvements were seen in overall function and need for oral or intrathecal medications. No hemorrhages or neurological complications of surgery were observed; however, the infection rate was significant at 14%. 

Vercueil et al. published their results with two patients suffering from PKAN who underwent bilateral DBS implantation in the ventral intermediate thalamic nuclei. With a follow-up time of 120 months, one of the patients demonstrated improvement of 26% in the BFMDS score. In the same year, Trottenberg et al. reported on one patient with tardive dystonia whose BFMDS score improved by 72% six months after beginning pallidal DBS.

Andews et al. performed a metaregression analysis of individual patient outcomes by reviewing results of pallidal DBS in 466 patients from 157 papers published through 2010. The subclassification of patients included 344 with primary dystonia, 10 with myoclonus dystonia, 19 with neurodegenerative dystonias, and 93 with secondary dystonia. They concluded that patients with primary dystonia, myoclonus dystonia, certain subtypes of heredodegenerative dystonia, and tardive dystonia have a >50% average improvement in dystonia rating following DBS. In patients with primary generalized dystonia, a multiple regression analysis revealed that lower baseline severity score, shorter duration of symptoms, and DYT1 positive status were all associated with a significantly higher likelihood of improvement. Patients with secondary dystonia and heredodegenerative disorders had a more varied response, making conclusions regarding predicted outcomes more difficult.

## 4. Conclusion

Dystonia is a syndrome of sustained muscle contractions producing abnormal postures, twisting, and repetitive movements. A variety of forms have been described, most of which are refractory to medical therapy alone. DBS has been used to treat several of these conditions but the success varies according to the type of dystonia. The treatment of dystonia with DBS is becoming increasingly common as evidenced by the rising number of published case reports on the rise. The most successful results appear to be in DYT1+ children or young adults. The question arises as to whether or not other genetic dystonias respond to GPi DBS. While patients with primary dystonia appear to have superior results compared to those with secondary forms of the disease, the latter patients, in some cases, may still be candidates for DBS. What is now needed is a randomized trial with long-term followup to identify who are the best candidates, when best to intervene with DBS, and where best to place the lead.
